# Courtship Ritual of Male and Female Nuclei during Fertilization in Neurospora crassa

**DOI:** 10.1128/Spectrum.00335-21

**Published:** 2021-10-06

**Authors:** Sylvain Brun, Hsiao-Che Kuo, Chris E. Jeffree, Darren D. Thomson, Nick Read

**Affiliations:** a Laboratoire Interdisciplinaire des Energies de Demain, CNRS UMR 8236, Université de Paris, Paris, France; b Manchester Fungal Infection Group, Division of Infection, Immunity, and Respiratory Medicine, University of Manchester, Manchester, United Kingdom; c Fungal Cell Biology Group, Institute of Cell Biology, University of Edinburgh, Edinburgh, United Kingdom; Universidade de Sao Paulo

**Keywords:** sexual reproduction, fertilization, nucleus, trichogyne, mating, live-cell imaging, fungi, *Neurospora crassa*, filamentous fungi

## Abstract

Sexual reproduction is a key process influencing the evolution and adaptation of animals, plants, and many eukaryotic microorganisms, such as fungi. However, the sequential cell biology of fertilization and the associated nuclear dynamics after plasmogamy are poorly understood in filamentous fungi. Using histone-fluorescent parental isolates, we tracked male and female nuclei during fertilization in the model ascomycete Neurospora crassa using live-cell imaging. This study unravels the behavior of trichogyne resident female nuclei and the extraordinary manner in which male nuclei migrate up the trichogyne to the protoperithecium. Our observations raise new fundamental questions about the *modus operandi* of nucleus movements during sexual reproduction, male and female nuclear identity, guidance of nuclei within the trichogyne and, unexpectedly, the avoidance of “polyspermy” in fungi. The spatiotemporal dynamics of male nuclei within the trichogyne following plasmogamy are also described, where the speed and the deformation of male nuclei are of the most dramatic observed to date in a living organism.

**IMPORTANCE** Using live-cell fluorescence imaging, for the first time we have observed live male and female nuclei during sexual reproduction in the model fungus Neurospora crassa. This study reveals the specific behavior of resident female nuclei within the trichogyne (the female organ) after fertilization and the extraordinary manner in which male nuclei migrate across the trichogyne toward their final destination, the protoperithecium, where karyogamy takes place. Importantly, the speed and deformation of male nuclei were found to be among the most dramatic ever observed in a living organism. Furthermore, we observed that entry of male nuclei into protoperithecia may block the entry of other male nuclei, suggesting that a process analogous to polyspermy avoidance could exist in fungi. Our live-cell imaging approach opens new opportunities for novel research on cell-signaling during sexual reproduction in fungi and, on a broader scale, nuclear dynamics in eukaryotes.

## INTRODUCTION

Fungi are living microorganisms that considerably impact human life. These adaptive eukaryotes have been shown to thrive in the damaged Chernobyl radioactive reactors (1), space (2), and extreme temperatures (3). Their impressive growth capacity is used for biotechnology and industrial purposes (4, 5). Fungal plant pathogens are responsible for the loss of billions of dollars in food security due to crop losses worldwide, while human fungal pathogens are a major health problem that threatens the lives of millions (6–9). However, our knowledge of whole reproductive life cycles in the fungal kingdom is only partial. Sexual reproduction is a key event in the fungal life cycle, where evolution and selection rely on its creation of novel and beneficial genetic combinations in the organism (10, 11). The fungal reproductive cycle relies on elaborate genetic regulatory systems as well as complex cytological events (12–14). Understanding reproduction provides means to mitigate fungal threats and enhance biotechnological processes.

In heterothallic model organisms such as the filamentous fungus Neurospora crassa and Saccharomyces cerevisiae yeast, sexual reproduction only occurs with opposite mating-type partners (*mat a* and *mat A* in N. crassa) (15). In N. crassa, Dodge identified the conidium as the male partner and the ascogonium as the female partner (16). The ascogonium is embedded within a multihyphal female structure called the protoperithecium (17). The ascogonium produces specialized hyphae called trichogynes to facilitate fertilization. Trichogynes are chemotropically attracted by a diffusible chemical signal emitted by conidia (18, 19). Trichogynes eventually fuse to their macro- or microconidial mate, allowing successful fertilization and development of the protoperithecium into a perithecium. Importantly, the nuclei from both parents do not undergo karyogamy within the trichogyne (20–22).

Trichogynes express G protein-coupled receptors (GPCRs) at their surface which respond to the pheromone signal emitted by conidia (23–29). In ascomycetes, the expression of both the pheromone and the GPCRs are under the control of the *mat* locus (13). Trichogynes, which are attracted by the pheromone signal bind to, coil around, and eventually fuse with male conidia, eventually establishing plasmogamy. Although the migration of male nuclei has never been observed, it is assumed that once within the female trichogyne, male nuclei (of the opposite sex mating type) migrate up the trichogyne to the ascogonium, where they proliferate together with female nuclei, eventually forming the dikaryotic bag (30). Karyogamy and meiosis take place once two nuclei of each mating type exit the dikaryotic bag to enter the ascogenous cell. Although fertilization and development of perithecia have been imaged in several ascomycete models, merely by electron micrographs (16, 20, 31–35), the actual behavior of male and female nuclei in this fundamental process has never been tracked in living cells.

In the lab, fertilization in N. crassa can be observed a few hours after inoculating the protoperithecia with a suspension of opposite mating-type conidia, making this fungus a well-suited experimental model to study fertilization (18–20). Here, using parental N. crassa strains in which nuclei were visualized with either the synthetic green fluorescent protein (H1-sGFP) (36) or the tdimer red fluorescent protein (H1-RFP) fused to the histone H1 protein, we achieved for the first time live-cell imaging of male and female nuclei during fertilization. Our observations highlight remarkable differences in the behavior of nuclei from different parental origins within the same cellular cytoplasm. Moreover, we imaged the astounding dynamism of male nuclei as they migrate by repeated stretching and contraction up the trichogyne, passing by immobilized female nuclei. Finally, our results highlight the intriguing signaling network controlling these movements of both male and female nuclei throughout fertilization. In particular, our observations led us to hypothesize that a system preventing the protoperithecium from being fertilized by several male nuclei in N. crassa may operate similarly to systems avoiding polyspermy in mammals (37).

## RESULTS

### Chemotropic growth of trichogynes.

**FIG 1:**
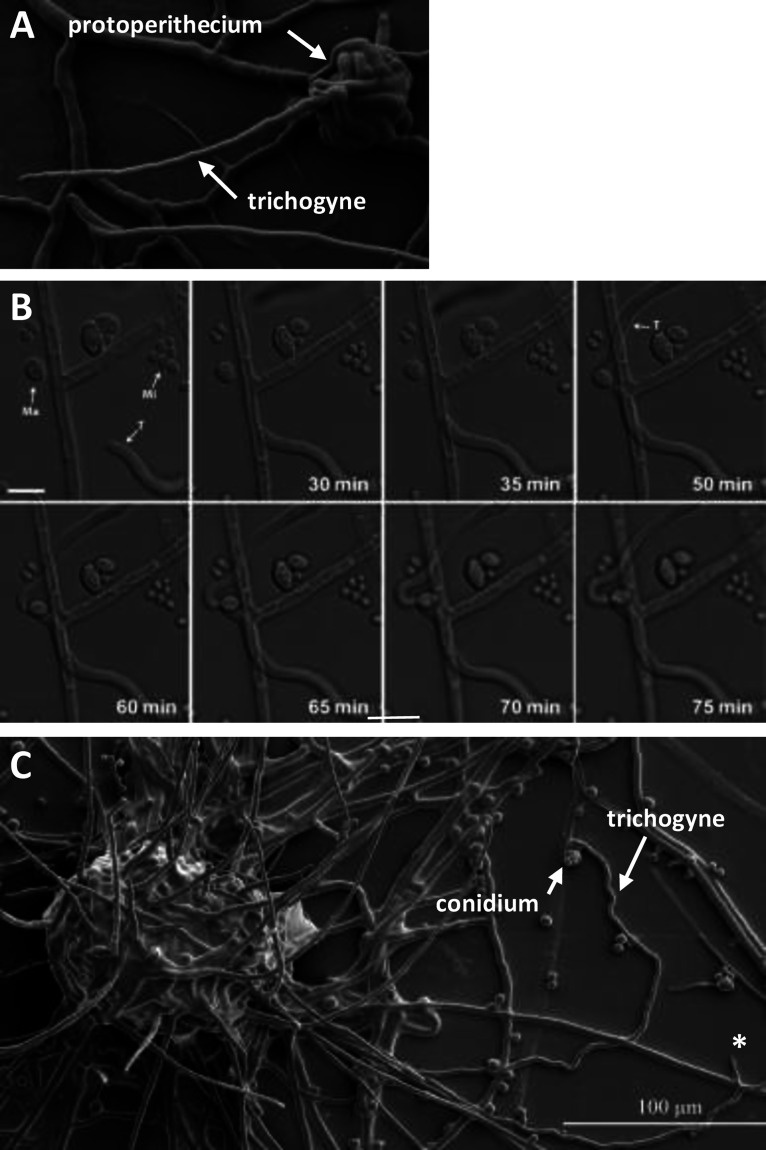
Trichogynes and chemotropic growth in N. crassa. (A) A SEM image of a young (3 days old) protoperithecium emitting a single trichogyne; scale bar = 10 μm. (B) Time-lapse sequence of the tropic growth of two trichogynes (T) toward one isolated macroconidium (Ma) while ignoring other macroconidia and microconidia (Mi). The trichogynes are not attracted to the microconidia in this field of view. Scale bar = 10 μm. (C) SEM image of a 5- 7-day-old protoperithecium, where one trichogyne has been attracted and coiled around a conidium. *, Probable trichogyne. Scale bar = 100 μm.

**FIG 2:**
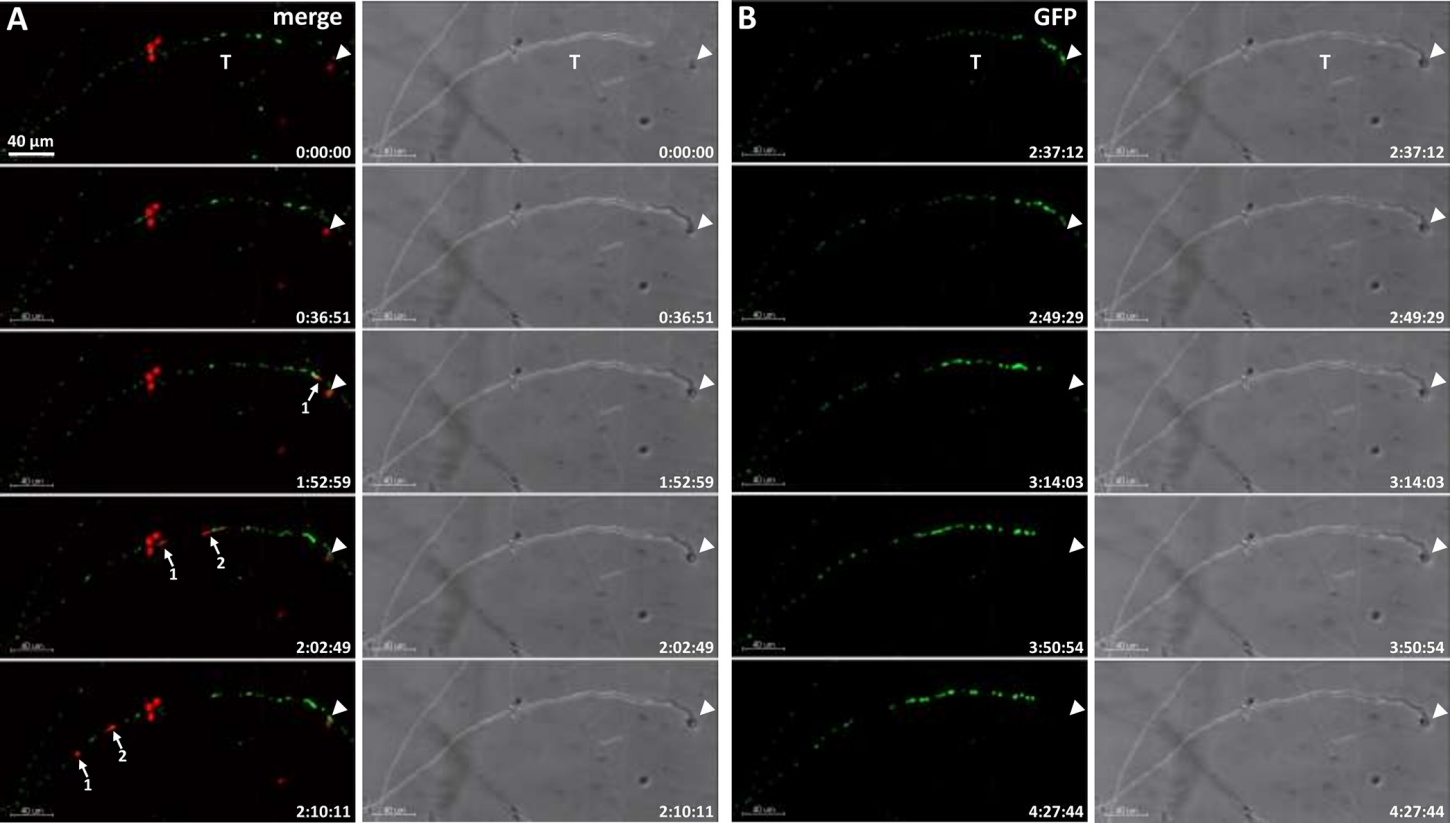
Time-lapse imaging of male and female nuclei before and after plasmogamy (Movie S2). Female *mat A H1-sGFP* was fertilized with *mat a H1-RFP* male conidia. (A) merged fluorescence and bright-field confocal images (maximum intensity projected Z-stacks) during the initial stages of fertilization. The female trichogyne bearing sGFP nuclei tropically grows to one macroconidium containing RFP nuclei (arrowhead). Two RFP male nuclei from the macroconidium (arrows 1 and 2) enter and migrate up the female trichogyne. (B) GFP fluorescence and bright-field confocal images (maximum intensity projected Z-stacks) during the later stages of fertilization after RFP male nuclei have left the field of view. Female sGFP nuclei undergo retrograde movement up the trichogyne from its coiled tip (arrowhead). T, trichogyne. Scale bar = 40 μm.

To visualize the fertilization process in N. crassa, we adapted existing experimental settings (18). This involved inoculating 1.5 by 1.5 cm agar blocks bearing 5- to 7-day-old protoperithecia producing “female” trichogynes with a freshly prepared (0- to 5-day-old) mixed suspension of “male” macro- and microconidia from the opposite mating type. After 6 h of inoculation, the agar blocks were inverted onto a micro-slide chamber containing a droplet of classic Vogel’s medium and imaged on either an inverted Nikon TE-2000 wide-field or Leica SP8x confocal microscope. Crosses were performed in both directions—♀ *mat A* × ♂ *mat a* as well as ♀ *mat a* × ♂ *mat A*. We observed no differences between the two cross types. Protoperithecia develop on mycelium during the stationary phase, where growing vegetative hyphae are rare. Within the mycelium network, trichogynes were identified via the following morphological features (Movies S1 and S2; all the movies are downloadable from https://figshare.com/s/e3c33f69ef0131ca6670 [Please do not play them directly online since they do not all play. Download them before playing them.]) reaching several hundred micrometers (Fig. 1, 2, and 6); exhibiting tropic and sinusoidal growth toward conidia (Fig. 1B and C and 2 and Movies S1 and S2) at a reduced extension rate of 1.1 ± 0.2 μm/min, compared to vegetative hyphae at the colony edge (67 μm/min) (38), and exhibited coiled growth around conidia. In addition to the observation that vegetative hyphae did not exhibit sinusoidal growth (data not shown), trichogynes were only distinguished from vegetative hyphae once they had coiled around a conidium of the opposite mating type. The coiling of the trichogyne around the conidium was the hallmark of “successful” fertilization (Fig. 1C, 4A, 5A, and 6). Although we only show here evidence for macroconidia, we also observed microconidia attracting trichogynes (data not shown) as previously shown (18, 19, 21, 22).

Since conidia are randomly spread on trichogyne-bearing agar blocks, the proximity of protoperithecia to individual conidia or groups of conidia varied. As a result, trichogynes from individual protoperithecia were observed to extend and fuse with conidia located between a few microns to half a millimeter from their site of origin. We observed very long trichogynes (>500 μm) from a protoperithecium fusing with a conidium located close to another protoperithecium. As observed in Fig. 1B, trichogynes do not necessarily fuse with the first conidium encountered but can instead be attracted to other conidia further away. Interestingly, an isolated singular macroconidium, in the vicinity of clusters of macroconidia and microconidia, attracted two trichogynes, suggesting heterogeneity in the conidial ability to attract trichogynes (Fig. 1B).

### Behavior of female nuclei in the course of fertilization.

We imaged *H1-sGFP* tagged female nuclei (i) during the tropic growth of trichogynes toward male conidia, (ii) after the trichogyne had coiled the conidium and received the first *mat a H1-RFP* male nucleus, and (iii) when male nuclei migrate up to the trichogyne base. The first contact between conidia and trichogynes was regularly observed 4 to 6 h after inoculation with the conidial suspension. Entry of male nuclei into trichogynes and protoperithecia occurred 6 to 8 h and 8 to 10 h after inoculation, respectively.

**FIG 3:**
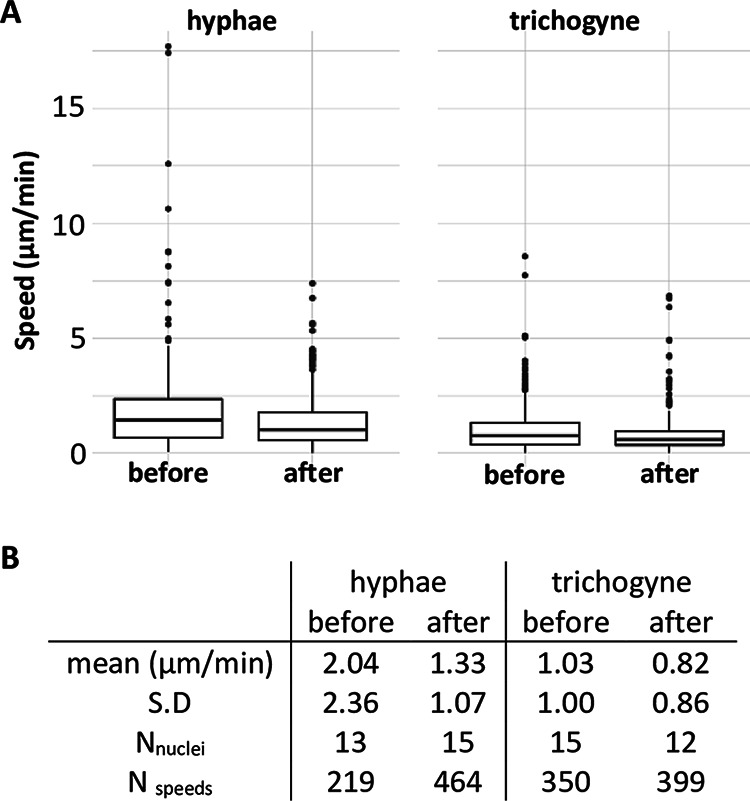
Female nuclear speeds before and after male nucleus entry into the trichogyne. Analysis of the time-lapse Movies S3a and b. (A) Plot; (B) numerical values. The speed of the green-labeled *H1-sGFP* female nuclei of the trichogyne and of the vegetative hyphae in the same field of view were measured at every time frame before (Movie S3a) and after (Movie S3b) entry of a male nucleus into the trichogyne. S.D, standard deviation; N_nuclei_, number of analyzed nuclei in the field of view; N_speed_, number of nuclei speeds measured. Results from the ANOVA (on log values) indicate that the effect of target site is significant (*P* < 0.001), with larger values in hyphae (1.56 ± 1.63, *n* = 683) than in trichogyne (0.92 ± 0.94, *n* = 749); likewise, the effect of time of observation is significant (*P* < 0.001), with larger values before (1.42 ± 1.73, *n* = 569) than after (1.10 ± 1.01, *n* = 863); these two effects are independent of one another as confirmed by the nonsignificant interaction (*P* = 0.431). Pairwise *t* tests with pooled SD indicate that all pairs of means were different at the 5% level.

Importantly, female and male H1 histone proteins, fused with either green (syntheticGFP; sGFP) or red (tdimerRed; RFP) fluorescent proteins, never mixed in the same nuclei. This enabled us to exclusively follow the GFP-female and RFP-male nuclei throughout the fertilization process in our imaging experiments. Furthermore, we never observed karyogamy (fusion of a male and a female nucleus) within trichogynes. Female nuclei were located and aligned throughout the trichogyne axis (Fig. 2 and Movie S2). During tropic trichogyne growth, GFP female nuclei displayed an overall anterograde movement at rates similar to the extension rate of the trichogyne. From Fig. 2 and Movie S2, upon trichogyne-conidium contact (time [*t*] = 36 min), it took 23 min for the trichogyne to complete conidium-coiling and cease growth (*t* = 59 min). Next, the first *H1-RFP* conidial nucleus took 53 min to enter into the trichogyne (*t* =1 h 52 min), followed by a second one 10 min later (*t* = 2 h 2 min), indicating that plasmogamy had occurred. Upon cessation of trichogyne growth at its targeted conidium, the anterograde movement of female nuclei switched to an oscillatory motion and then became visually immobilized 4 min before the entry of the first male nucleus (*t* = 1 h 48 min). Nucleus speed measurements revealed that female nucleus movements did not fully stop but were reduced (Fig. 3). For the sake of simplicity, we will refer to this phase as female nuclear immobilization. These female nuclear dynamics suggest stepwise signaling events anticipating the entry of the male nucleus. Female nuclei remained immobilized while male nuclei migrated up the trichogyne. Finally, 56 min after the first male RFP nucleus entered the trichogyne, all immobilized female GFP nuclei synchronously began moving backward (retrograde movement) up the trichogyne (*t* = 2 h 44 min). These female nuclear dynamics were consistent with every fertilization event analyzed (*n* = >10). We quantified female nucleus mobility before and after entry of male nuclei into trichogynes for at least one fertilization event (Fig. 3). By tracking nuclei in Movies S3a (before entry) and S3b (after entry), we measured the speed of GFP female nuclei within trichogynes after trichogyne growth had ceased at the conidium and after female nuclei were immobilized. These dynamics were compared to the mobility of female nuclei located in surrounding hyphae, likely vegetative ones since initial nucleus behavior in these hyphae were different (Fig. 3 and Movies S3a and b).

The average speed of hyphal female nuclei was significantly higher (2.04 ± 2.36 μm/min) than that of trichogyne female nuclei (1.03 ± 1.00 μm/min; Fig. 3; *P* < 10^−3^) prior to male nucleus entry. Strikingly, the speed of both hyphal and trichogyne female nuclei significantly decreased after male nucleus entry, to 1.33 ± 1.07 μm/min (*P* < 10^−3^) and 0.82 ± 0.86 μm/min (*P* < 10^−3^), respectively. These data confirmed the immobilization of female nuclei observed immediately prior to entry of male nuclei into the trichogyne. Note that the standard deviation (SD) values highlight the variations of individual nuclear movements at each time frame.

The shape of female H1-sGFP fluorescent nuclei in both the analyzed hyphae and in the trichogyne changed from ovoid to more rounded during their immobilized phase, as observed in Movies S3a and b, and reverted to ovoid at the resumption of movement (end of Movie S5). During the latter phase (Movie S5), the recovered mobility in female nuclei followed by their retrograde migration up the trichogyne was exemplified by the behavior of the nucleus, close to the conidium in Movie S5 (the top one). This exemplar nucleus became pear-shaped while being pulled in the retrograde direction. These data describe for the first time the dynamic nature of resident female nuclei within trichogynes during sexual fertilization in N. crassa. Moreover, the signal that triggers female nuclear immobilization is not restricted to the trichogyne subjected to plasmogamy, suggesting a possible diffusible signal, which can pause the nuclear dynamics of this filamentous fungus.

### Nuclear morphology and movement of residual conidial male nuclei.

**FIG 4:**
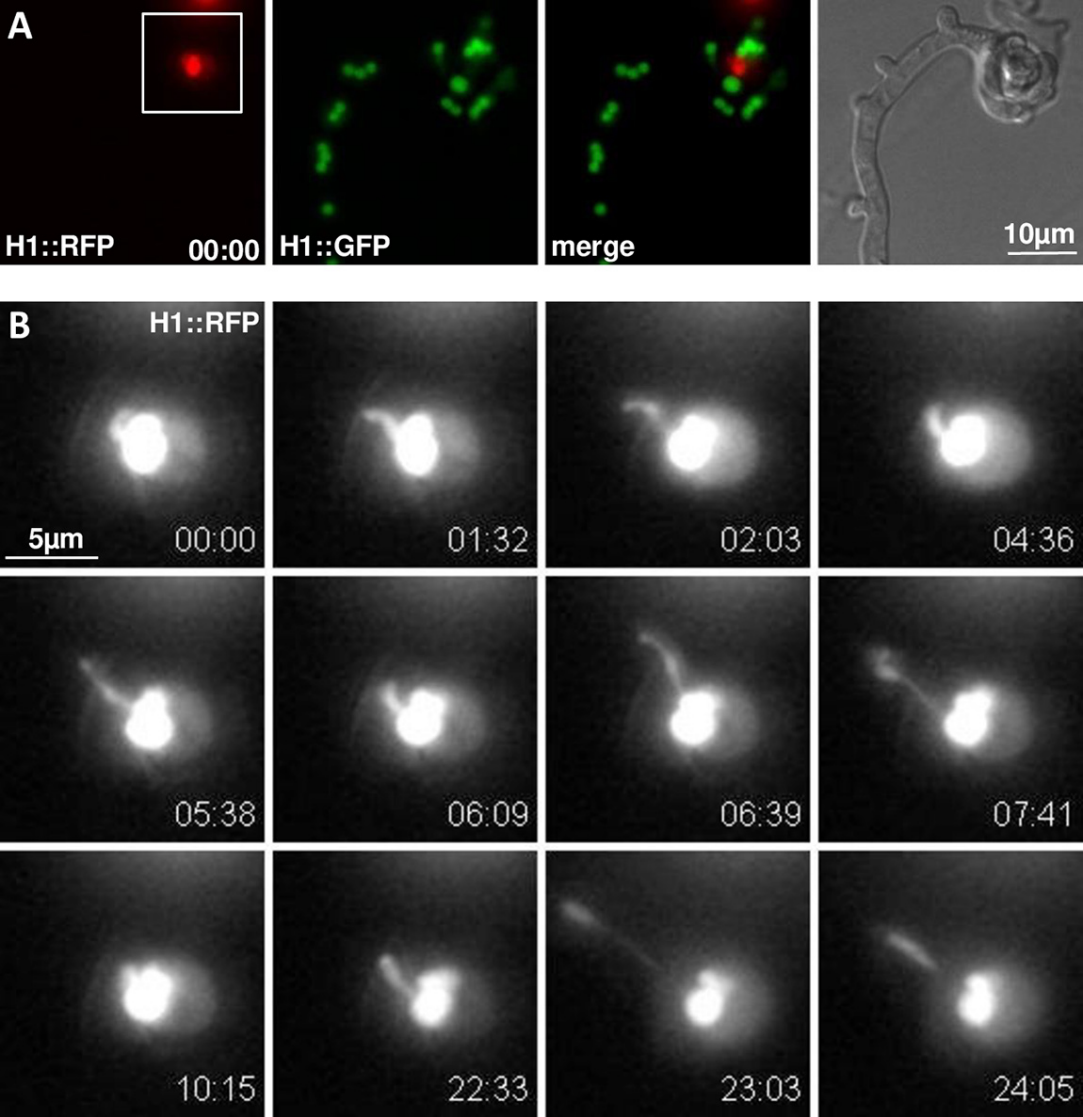
Time course of male nuclear mobility within the conidium upon fertilization (Movie S4). (A) Wide-field fluorescence and bright-field images of *H1-sGFP* female trichogyne fertilization by an *H1-RFP* conidium. (B) Higher magnification of the residual RFP conidial nuclei (box in A) after the first nucleus entered and migrated up the trichogyne (not shown). The imaged residual nucleus stretched and moved around within the conidium but failed to migrate up the trichogyne. Time scale = min:sec. Scale bars = 10 μm in panel A and 5 μm in panel B.

**FIG 5:**
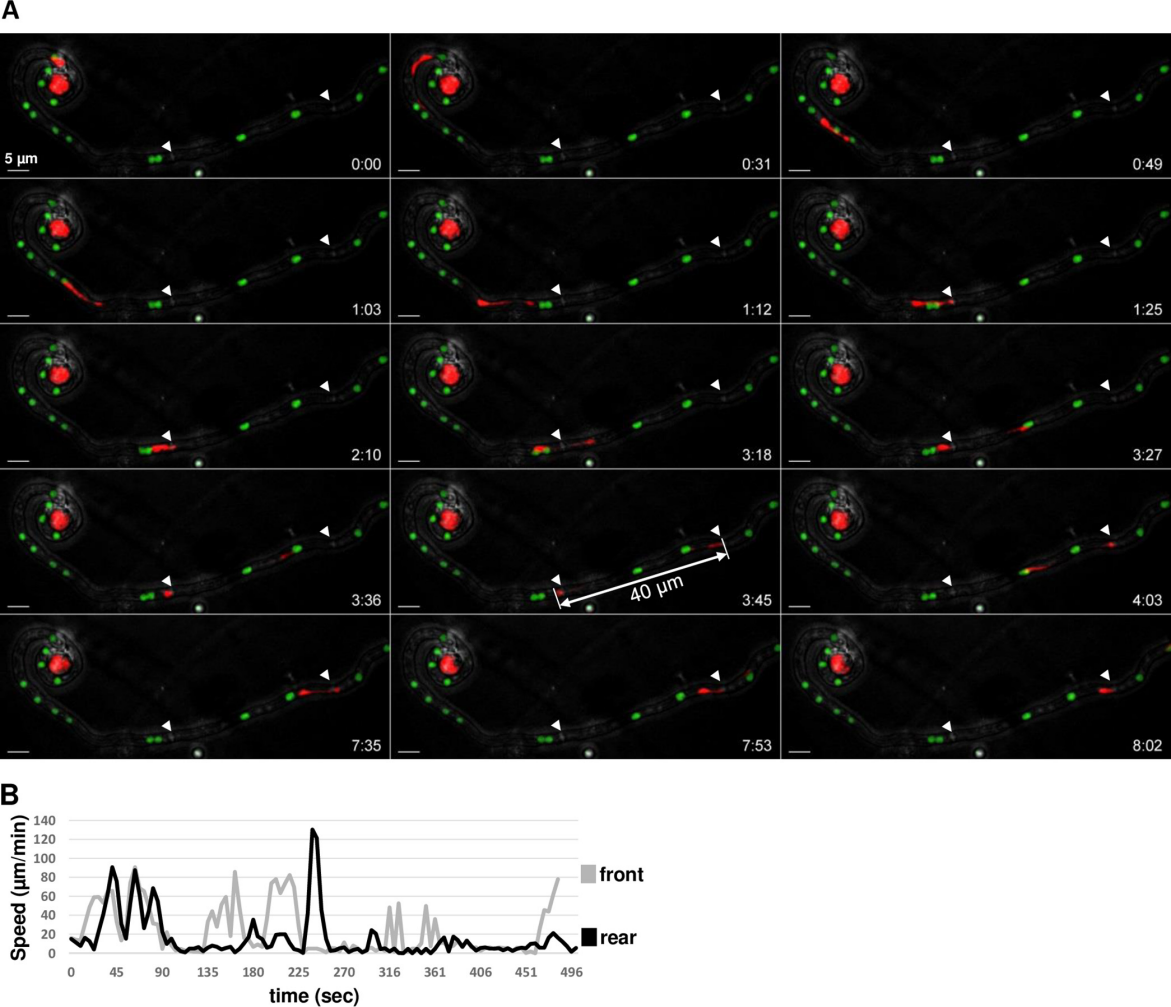
Time course of male nucleus movement across the trichogyne (Movie S5). (A) Merged confocal images (maximum-intensity projected Z-stacks) of the fertilization of a *mat A H1-sGFP* female trichogyne by a *mat a H1-RFP* conidium. The RFP nucleus enters and migrates up the female trichogyne successively, stretching and contracting in an inch-worm-like manner. The remarkable stretching (40 μm) of the male nucleus crossing the first septa (arrowheads) is indicated. Scale bar = 5 μm. (B) Velocity analysis of the exemplar stretching RFP nucleus at the front (gray) and rear points (black).

Using the protocol described previously, we aimed to image *mat a H1-RFP* male nuclei at the onset of their entry into female *mat A H1-sGFP* trichogynes. At 4 to 6 h after inoculation with conidia of the opposite mating type, scarce trichogynes coiling around conidia (fertilization events) were observed on inoculated agar blocks. In order to image early steps of male nuclear migration, image acquisitions were started as soon as coil fertilization hallmarks were found. This way, we observed that male RFP nuclei were highly mobile, rotating while still enclosed within the conidium (Fig. 4 and 5; Movies S4 and S5). Eventually, these rotating movements accelerated until the nucleus (sometimes one of several within the macroconidium) started to repetitively elongate toward the entry of the trichogyne before finally migrating up the trichogyne. These sequential morphology changes were reproducibly observed prior to the onset of male nuclear migration.

However, regarding the nucleus imaged in Fig. 4 and the second nucleus remaining in the conidium (after migration of the first one) in Fig. 5 (Movies S4 and S5), both failed to enter the trichogyne. Both observations of male nuclei failing to engage migration were representative of other live-cell recordings, where fluorescently imaged male nuclei did not enter the trichogyne (data not shown). This led us to hypothesize that the lack of male nuclear migration during live-cell imaging may be due to phototoxicity and that real-time frame-rate acquisitions had to be limited prior to migration observation.

### The inch-worm movement of male nuclei.

To minimize phototoxicity to the fertilization process, fertilization events were checked infrequently in order to preserve the integrity of subsequent live-cell imaging of the male nuclear migration. This extra care allowed observation of several male nuclear migration events into trichogynes. The trichogyne entry process was not straightforward; male nuclei do not directly enter the trichogyne but, instead, paused frequently prior to entry. It was frequently observed that individual male nuclei stretched and retracted in and out of the trichogyne several times without leaving the conidium (data not shown). This “inch-worm” nuclear movement is illustrated in Fig. 5 and Movie S5, where the RFP male nucleus moved in an “inch-worm” manner, repetitively stretching and contracting while migrating up the trichogyne. Stretching was so dramatic that male nuclei seemed sometimes split into two (Fig. 5; from 3 min 18 s to 4 min 3 s; Movie S5). The way male nuclei stretched suggested that the front is submitted to pulling mechanical strain during migration (see Discussion). Nuclear elongation was especially striking when the male nucleus passed through septa. In Fig. 5, the male nucleus passed through the first septum (Fig. 5; *t* = 2 min 10, s to *t* = 4 min 3 s) and, remarkably, stretched to a maximum length of 40 μm (*t* = 3 min 27 s; Fig. 5), where its apical end was pulled while its rear end was “stuck” at the septal pore. To understand this process more, we measured the speed of each nuclear apex (front and rear) in the movie. The maximum speed measured for the front side was 85 μm/min, while the maximum speed of the rear side reached 130 μm/min (Fig. 5B). These data highlight the remarkable spatiotemporal dynamics and nature of migrating fungal nuclei during fertilization in N. crassa.

### Fertilization of the protoperithecium by male nuclei.

**FIG 6:**
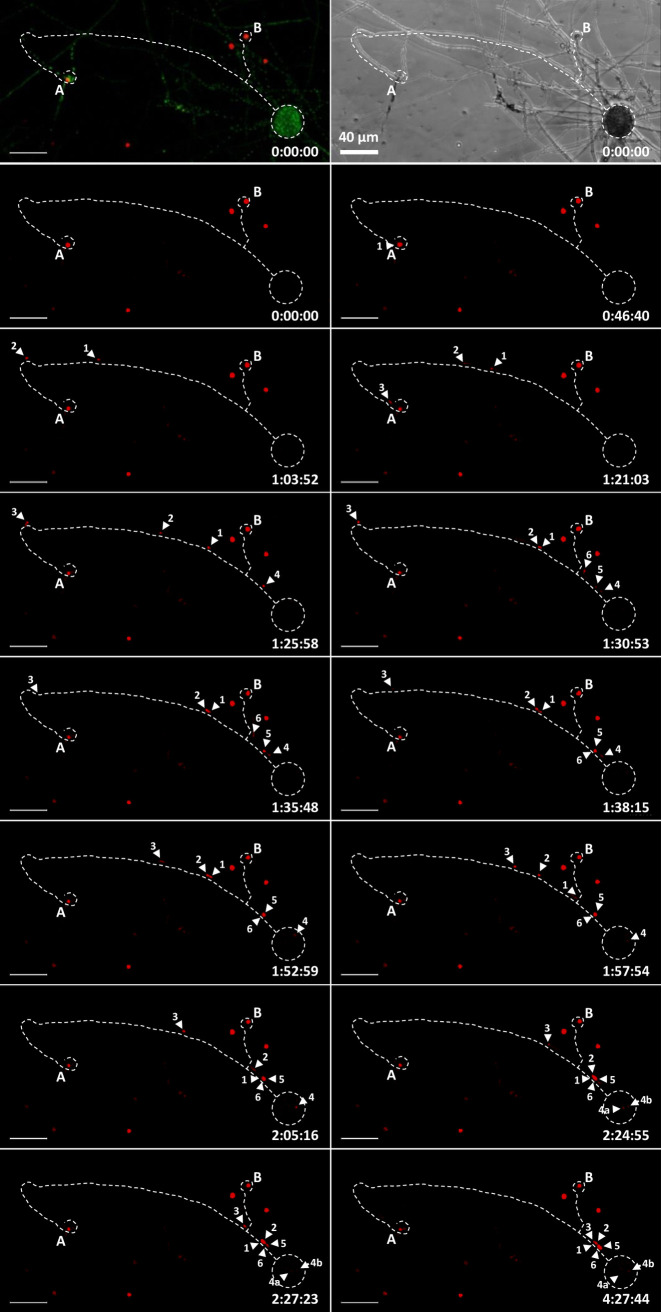
Time course of the fertilization of a protoperithecium by a single male nucleus (Movie S6). Confocal microscopy of the fertilization of an H1-sGFP female with H1-RFP conidial suspension. The first row shows merged GFP female nuclei and RFP male nuclei with a bright-field representation at *t* = 0 min. Subsequent time-lapse RFP images contain a dotted trace of the trichogyne and protoperithecium. Two conidia (A and B) attracted and fused with a branched trichogyne emitted by the protoperithecium at the bottom-right corner. Three nuclei (1, 2, and 3) from conidium A and three nuclei (4, 5, and 6) from conidium B successively enter the trichogyne. Nucleus number 4 enters the protoperithecium core and eventually divides (4a and 4b). Scale bar = 40 μm; time scale = h:min:sec.

After trichogyne entry, the conidial nuclei travel to the protoperithecium, where the next steps of sexual reproduction, such as karyogamy, meiosis, and ascosporogenesis, take place. In N. crassa, macroconidia are typically multinucleate. We reproducibly observed that fertilization by macroconidia led to the discharge of several nuclei from a single conidium into a trichogyne (i.e., three nuclei per conidium in Fig. 6, 2 nuclei in Fig. 2, and 2 nuclei in Movies S7 and S8). Furthermore, we observed that trichogynes can branch and that those branches are capable of binding, coiling, and fusing with at least two conidia (A and B; Fig. 6 and Movie S6). In the trichogyne of Fig. 6, male nuclei were tracked from the conidia to the protoperithecium over a distance of up to 574 μm (Fig. 6; conidium A). Three male RFP nuclei entered the trichogyne from conidium A in 35 min (Fig. 6, labeled 1, 2, and 3; from *t* = 46 min to *t* = 1 h 21 min), followed by three additional nuclei from conidium B, which were released in only 6 min (Fig. 6, labeled 4, 5, and 6; from *t* = 1 h 25 min to *t* = 1 h 31 min). By carefully tracking the different male nuclei within the trichogyne, we determined that the nucleus entering the protoperithecium was nucleus 4 from conidium B (Fig. 6; *t* = 1 h 53 min). Strikingly, although the remaining male nuclei continued their migration up the trichogyne, all of them stalled and accumulated upstream of the now-fertilized perithecium (Fig. 6; *t* = 4 h 24 min; Movie S6). It was also the case in Movie S7, where two conidial male nuclei migrated up the trichogyne and only one entered the protoperithecium. Accordingly, immobile RFP male nuclei in trichogynes were frequently observed late in the experiment (between 10 and 14 h), where presumably, nuclei were somehow blocked from migrating after protoperithecium fertilization (data not shown). Note that the two full fertilization events imaged (Fig. 6, Movies S6 and S7) were the only recordings where we tracked multiple male nuclei migrating into a trichogyne from plasmogamy to entry into a protoperithecium. These live-cell recordings led us to hypothesize that entry of a first male nucleus into a protoperithecium inhibits entry of the following nuclei.

**FIG 7:**
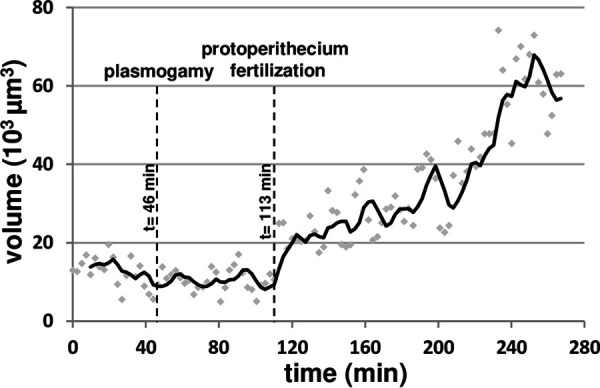
Volume increase of protoperithecium after its fertilization by entry of one male nucleus. Analysis of segmented images of *H1-sGFP* fertilization by *H1-RFP* conidial suspension in Movie S6 (Fig. 6). The green signal of the *H1-sGFP* nuclei composing the protoperithecium was segmented in order to evaluate the volume of the perithecium in the course of fertilization. The time of entry of the first *H1-RFP* male nucleus into the trichogyne (plasmogamy) and the time of entry of the nucleus number 4 into the protoperithecium (protoperithecium fertilization) are indicated. Dots, individual data points; black line, moving average.

A second important feature provoked by entry of male nuclei into prothoperithecia was the quantified increase of volume of the latter (Fig. 6; from *t* = 1 h 53 min; Fig. 7). Finally, we detected a second RFP focal signal in the core of the protoperithecium twice (Fig. 6; *t* = 2 h 25 min to 4 h 27 min; Movie S7; 4 h 22 min 52 sec). Careful analysis of the images did not indicate any entry of any of the remaining nuclei. Thus, the appearance of this second RFP focal signal 32 min after initial protoperithecium entry in Fig. 6 (Movie S6) may indicate that this male nucleus 4 has divided. However, we cannot exclude that the appearance of a second focus within the protoperithecium may be due to the stretching of the nucleus. These data suggest that entry of male nuclei into protoperithecia may trigger at least two signals, a first one in order to avoid polyspermy by blocking multiple fertilization of the protoperithecium and a second signal committing the protoperithecium into a growing and developing perithecium.

### The orienteering race of male nuclei up the trichogyne.

Another astonishing behavior of conidial male nuclei is their capacity to rapidly switch between anterograde and retrograde movement during trichogyne migration (Movies S8 and S9). Moreover, trichogynes with branches had male nuclei switching direction at T-junctions, illustrated in Movie S8 (the RFP male nuclei are colored in yellow in this movie), where the first male nucleus initially migrated right but then reversed and migrated left toward its intended pathway to the protoperithecium. This direction switch of male nuclei at T-junctions has been observed several times in independent experiments (data not shown). These observations led us to conclude that male nuclei are endowed with the capacity to change direction in the course of migration and to perhaps follow a yet unknown signal toward the protoperithecium.

An interesting feature of trichogynes is their ability to fuse with several conidia and, as a consequence, to allow the discharge of nuclei belonging to different conidia (Fig. 6 and Movie S6). It is interesting to note that nuclei from conidium A stopped migration, especially nucleus 1, remaining immobile for 23 min (from *t* = 1 h 25 min 58 s to *t* = 1h 52 min 59 s), while the nuclei from conidium B were released into the tricogyne. We measured the average speed of nuclei 1 and 2 before the pause and compared it to their average speed all along the whole trichogyne. Before the pause and across the first 420 μm of migration, the speeds of nuclei 1 and 2 were 10.9 and 12.3 μm/min, respectively, compared to 6.9 and 8.0 μm/min, respectively, for the entire migration of both nuclei. This indicated that the speed of male nuclei is not constant during their migration all along the trichogyne and that migration of some male nuclei may interfere with the migration of other nuclei within the same trichogyne.

## DISCUSSION

Sexual reproduction in fungi and fruiting body development have attracted the interest of researchers for centuries (39). Imaging these structures under non-live-cell imaging conditions, such as electronic microscopy, has highlighted the extraordinary complexity of fruiting bodies (31–34). However, the lack of live-cell biology has been an obstacle to fully understanding sexual reproduction from the initial plasmogamy of sexual cells to the final production of sexual spores. In previous *Neurospora* studies, following the work of B. O. Dodge, who identified the sexual reproductive organs in the *Neurospora* genus, Backus in 1939 observed the fertilization of trichogynes by conidia and described it through incredibly precise drawings (15, 20). In particular, Backus observed that trichogynes could be branched and were able to bind to and coil around (presumably fuse with) several conidia of the opposite mating type (20). Almost a century after these pioneering studies, using modern live-cell imaging technologies, we are able to image live conidial fertilization. Through live-cell imaging, we visualized the stages of N. crassa ontogeny for the very first time, encompassing the migration of male nuclei along the entire trichogyne length from their parental conidia to the ascogonium at the core of protoperithecia. Our experimental system contained male and female partners differentially bearing H1-sGFP or H1-RFP tagged nuclei. Importantly, we never observed nuclei sharing red and green fluorescent protein, implying that both these reporter proteins (H1-sGFP and H1-RFP) did not mix once nuclei bearing each of these transgenes were together within the same trichogyne cytoplasm. This property makes our experimental model well suited to describe and demonstrate the striking opposite behaviors of male and female nuclei within the trichogyne and confirmed that karyogamy does not take place right after plasmogamy within the trichogyne in this fungus (21, 22, 30).

During trichogyne growth, resident female nuclei undergo anterograde movement at a comparable speed to the trichogyne. Whether this movement is supported by cytoplasmic flow as in vegetative hyphae and whether female nuclei divide during trichogyne growth remain to be addressed (40). Upon cessation of trichogyne growth at the male conidium, female nuclei occupy the entire length of the trichogyne and are clustered regularly, especially at the trichogyne tip. After contact between the trichogyne and the conidium, the female nuclei only show oscillatory movements. In a strikingly concerted manner, movements of female nuclei stop right before entry of the first male nucleus into the trichogyne and eventually resume, showing female nuclei migrating backward toward the protoperithecium. Identifying what controls female nuclear movements during fertilization will be a great challenge. The first clues are given by the observation of female nuclear shape during the different steps described above. Indeed, female nuclear shape changes from oval or pear-shape before entry of male nuclei to round during the immobilization phase and then oval or pear-shaped when they move again. These changes may be reminiscent of modifications in the interaction between these nuclei and the cytoskeleton. In filamentous fungi, the microtubule (MT) cytoskeleton, as well as its associated molecular motors, i.e., the kinesins and the dynein-dynactin complex, have been involved in finely tuned movements such as shape changes, oscillatory movements, nuclear division, and nuclear distribution in hyphae. In particular, both changes of shape and oscillatory movements are affected when cells are treated with MT-depolymerizing drugs as well as in the *ropy* mutants altered for the dynein-dynactin complex (36, 41–44). Moreover, nuclei in the *ropy* mutants and in the *kin-1* mutant (altered for the major molecular motor Kinesin-1) have a rounded shape, in contrast to the oval or pear shape of nuclei in wild-type cells. Therefore, it is likely that the oscillatory movement of female nuclei within the trichogyne might involve the MT cytoskeleton and the kinesin/dynein molecular machinery. We speculate that the change of shape of female nuclei together with their immobilization may be reminiscent of momentary loss of interaction of these nuclei with the MT cytoskeleton machinery during the time when male nuclei migrate. In addition, we have observed that the immobilization of female nuclei may not be trichogyne-autonomous, since it was detected in surrounding hyphae. Altogether, these data led us to hypothesize that a diffusible signal that triggers immobilization of female nuclei is produced at the onset of male nuclear entry into the trichogyne.

In contrast to the resident female nuclei, male nuclei have to migrate up the trichogyne in order to reach the ascogonium in the core of the protoperithecium. Here, this assumption has been proven correct via the tracking of male nuclei all along their migration across the trichogyne until the core of the protoperithecium, presumably the ascogonium. We observed that several male nuclei could enter a single trichogyne, raising the question of the poly-fertilization of protoperithecia. The possibility for more than one male nucleus to fertilize a single protoperithecium has genetically been addressed in N. crassa (45–47). Sansome was the first to demonstrate that ascospores from a single perithecium could originate from more than one pair of parental nuclei. In 1960, Weijer (47) showed that 23% of the perithecia contained rosettes initiated by more than one male nucleus when a heterokaryotic male parent was used. However, Nakamura et al. (45) found that only 2% (60/2,770) of perithecia display mosaic rosettes accounting for fertilization by two or more different male nuclei when a mix of genetically different conidia is used. This shows that when using this more “natural” method (very similar to our fertilization conditions), fertilization of a single protoperithecium by more than one male nucleus is a rare event.

Here, we show for the first time that several male nuclei can indeed enter a single trichogyne and that they can originate from different conidia. Furthermore, we often observed branched trichogynes, suggesting that fertilization of a protoperithecium by more than one male parent is feasible, albeit likely rare (45). However, in the two cases for which we tracked several male nuclei migrating as far as the protoperithecium, we observed that only one male nucleus penetrated the core of the protoperithecium, while the following nuclei within the same trichogyne remained outside. Although further experiments will be required to confirm these observations, we speculate that entry of the first male nucleus into the protoperithecium may inhibit entry of the following ones. If such a blockage exists, it may be leaky, thus facilitating poly-fertilization of protoperithecia in rare cases (45). This blockage mechanism in the fungus N. crassa may be reminiscent of the mechanism in mammals which avoids “polyspermy.”

Although we ignore how male nuclei find the way to the protoperithecium in a branched trichogyne, we observed male nuclei first making a hesitant turn at T-junctions and then reversing to take the other branch, likely the correct path to the protoperithecium. This highlights that male nuclei can rapidly change direction in a trichogyne (moving back and forth in a retrograde versus an anterograde movement) and that the migration path is not unique, nor is its signaling precisely defined. We speculate that this signaling may regulate the tethering of male nuclei to molecular motors but, also, the choice of the appropriate molecular motor to move in the anterograde or the retrograde direction.

The most striking features observed in our attempts to characterize the behavior of nuclei in the course of fertilization are indubitably the ones related to male nuclei. Male nuclei undergo a series of morphological changes within the conidium at the onset of migration. Since we could not observe when cell fusion (plasmogamy) between trichogynes and conidia occurs, how these morphological changes in the nuclei synchronize with plasmogamy remains to be addressed. Next, male nuclei undergo initial stretching upon leaving the conidium. Male nuclei then migrate up the trichogyne, repetitively stretching and contracting in an inch-worm-like manner. Generally, the migrating nuclei harbor a compact shape before encountering septa and elongate dramatically while passing through. This stretching can be as extreme as 40 μm in length, around 20 times the size of standard nuclei (2 μm), shedding light on two important features of migrating male nuclei—the extraordinary plasticity of the nuclei and the strong pulling force applied to the nucleus. This plasticity is particularly striking when the rear of the nuclei remains blocked at septa while, in the meantime, the front is repetitively pulled, leading to repetitive stretching (Movie S5). In the end, like a rubber band stretched and released at one side, the rear of the nucleus eventually joins the front in a movement exerting one of the highest speeds ever measured for nuclei in cells (130 μm/min). We propose that the force moving nuclei in the trichogyne is likely a pulling force applied to the front of the nucleus and that the rear follows by a spring effect. Since male nuclei can rapidly change direction and move back and forth, this implies that the leading “front” of nuclei can become the “rear” and vice-versa almost instantly.

Regarding the male nuclear velocity, its versatility, and the fact that during this impressive mobility, female nuclei remain immobilized, we exclude the cytoplasmic flow to be responsible for male nuclear movements. Hence, we suppose that the movements of male nuclei are undertaken by molecular motors and the cytoskeleton (41). Further studies are required to determine the role of the microtubules and actin, as well as the role of their cognate molecular motors, i.e., myosin versus dynein/dynactin and kinesin, respectively, in the movement of male (but also of female) nuclei, within the trichogyne during sexual reproduction.

Nuclear movements and deformation have been studied in many organisms (plants, animals, and fungi) and in many contexts (during development, sexual reproduction, hematopoiesis, metastases, migration, etc.). No such deformation or speeds have been reported in plants or animals, where one the highest speeds measured was 16 μm/min for the male pronucleus during fertilization in *Xenopus* (reviewed in reference 48). Interestingly, high nuclear speeds have been reported in N. crassa during hyphal growth (40). At the hyphal apex, nuclei migrate at a speed of 8 to 9 μm/min, principally propelled by bulk flow. These nuclei also show very rapid and minute anterograde/retrograde movements that can reach 74.4 μm/min for anterograde movements in the wild-type and even 222 μm/min for retrograde movements in a *ro-1* dynein mutant (40). These data show that N. crassa is endowed with a molecular machinery enabling very rapid nuclear movements, and it is likely that a similar machinery is involved in rapid male nuclear movements during their migration in trichogynes.

The difference in behavior of male and female nuclei inside the same fungal cell cytoplasm raises the question of the identity of both types of nuclei. Since heterothallic fungi can only reproduce by sexual reproduction with individuals of the opposite mating type, it is assumed that the mating type locus is the primary determinant of the male and the female nuclear identity (13). In particular, *mat* loci regulate the expression of the pheromones and their cognate GPCRs, which are both involved in the chemotropic interplay between conidia and trichogynes. Basically, binding of the pheromone produced by a conidium to its cognate receptor at the trichogyne membrane activates the GPCR signaling cascade responsible for the chemotropic growth, the coiling, and ultimately, the fusion with conidia of the opposite mating type only (25, 26, 28, 29). It will be of great interest to test whether the *mat* locus and the pheromone/GPCR pathway are involved in the control of male and female nuclear movements during sexual reproduction in N. crassa.

Through modern live-cell imaging, our work humbly aimed to answer questions about nuclear fate in the course of fertilization as old as the discovery of sex in fungi (39). However, our study broadens the perspectives beyond the simple study of fertilization by unravelling unexpected behavior of both male and female nuclei. This live-cell imaging approach to studying fertilization in the model ascomycete N. crassa raises new questions. What are the regulation pathways that control immobilization of female nuclei? What is the machinery that pulls male nuclei during migration? What signal guides male nuclei during migration? Is there a polyspermy avoidance, and how is it set up? How does male nuclear entry into protoperithecia trigger perithecial development? What defines nuclear identity? On top of this, the dramatic deformation of male nuclei during migration within the trichogyne and the amenability of model fungi such as N. crassa for live-cell biology and molecular genetics studies make fertilization in N. crassa a new system to use to study the effect of physical forces and the deformations they create on nuclei. Understanding how physical forces and constraints applied to nuclei modify the whole nucleus organization and trigger gene expression switches is an emerging area of research (49, 50). The number of human pathologies primarily due to failure in mechanotransduction of signals is constantly increasing (51), and recent data show that extreme deformation of nuclei in metastatic cells during migration has a priming effect in aggressiveness of these malignant cells (52). Therefore, the study of the plasticity of the male nucleus and of its chromatin structure during sexual reproduction in N. crassa offers a unique opportunity to unravel how extreme deformations of nuclei can modulate genome structure and gene expression in eukaryotes.

## MATERIALS AND METHODS

### Strains, culture conditions, and production of conidia.

The Neurospora crassa strains used in this the study were wild-type strains *74-OR23-1V A* (FGSC no. 2489) and *ORS-SL6 a* (FGSC no. 4200), N2282 *mat A his-3^+^*::*Pccg-1-hH1^+^-sGFP* (*H1-sGFP* in the article), N2283 *mat a his-3^+^*::*Pccg-1-hH1^+^-sGFP*, and *mat a his-3^+^*::*Pccg-1-hH1^+^-tdimerRed* (*H1-RFP* in the manuscript). Construction of the *H1-RFP* strain (no. 263 in the Nick Read collection) was conducted as follows: a plasmid expressing an N-terminal tdimerRed fluorescent protein (RFP) fused to histone H1 was constructed by inserting the N. crassa *hH1* gene, amplified from pMF280 (36) with primers hH1SpeF (5′-GCCACTAGTATGCCTCCCAAGAAGACCGAG-3′) and hH1XbaR (5′-GCCTCTAGATTGCCTTCTCGGCAGCG-3′) into pMF331 (53) digested with SpeI and XbaI. The resulting plasmid, pMF360, was transformed into N623 (*mat A his-3* [36) as previously described (54). Positive transformants were selected on minimal medium and screened for expression of tdimerRed-H1 (H1-RFP in the article) under a dissecting fluorescence microscope as previously described (53). A transformant (NMF138) was crossed with N3011 (*mata his-2*; *mus-51*::*bar+*) to generate homokaryotic progeny. One *mat a* progeny was called strain 263 in the Nick Read collection.

Strains were maintained and grown on solid classic Vogel’s minimal medium with 2% sucrose at 27°C. Conidia were harvested from 4- to 5-day-old cultures grown at 25°C on Vogel’s medium with constant light, filtered with Miracloth (Merck Millipore), washed in TS (0.05% Tween 80/9% NaCl/sterilized water), and stored in TS at 4°C for up to 5 days.

### Fertilization procedure.

Petri plates containing 2% agar (tap water) solid medium were prepared and inoculated with the female strain (*mat a* or *A*) and incubated for 5 to 7 days until protoperithecia developed. A 2- to 3-cm^2^ agar plug harboring protoperithecia was cut and transferred to an empty petri plate. This sample was inoculated with a 1 to 2.10^6^ conidia · Ml^−1^ suspension of the “male” partner of the opposite mating type. Conidial suspensions were prepared the same day as the inoculation or kept at 4°C in TS before use (up to 5 days). After 4 to 6 h of incubation at 25°C in a moisture chamber, the sample was mounted inverted onto a droplet of Vogel’s medium in a 2-well μ-Slide ibidiTreat chamber and inspected for fertilization. Alternatively, after the initial four hours of incubation at 25°C, the sample was kept at 4°C overnight and mounted the next day in the microscopy chamber. In both cases, migrating male nuclei were observed after 2 to 5 h of further incubation at room temperature.

### Low-temperature scanning electron microscopy (LTSEM).

For Fig. 1A and C, low sucrose agar (LSA) plates overlaid with sterile cellophane (525-gauge uncoated Rayophane; A.A. Packaging, Preston, UK) were used for sample preparation. Fig. 1A shows 3-day-old culture of the wild-type *mat A* N. crassa strain (74*A*). Figure 1C shows the 7-day-old wild-type (74a) N. crassa strain inoculated with wild-type *mat A male* conidia (74A) (14 h of incubation after inoculation). Cellophane rectangles carrying the specimen were cut out and attached to the surface of a cryospecimen carrier (Gatan, Oxford, UK) with a thin layer of Tissue-Tek OCT compound (Sakura Finetek, Torrance, CA) as an adhesive and cryofixed by plunging into subcooled liquid nitrogen. The specimen carrier was transferred under low vacuum to the cold stage of a Gatan Alto 2500 cryo-preparation system at −180°C and then to the cold stage of an S4700II field emission scanning electron microscope (Hitachi, Wokingham, UK), where it was warmed to −80°C under continuous visual observation until any surface ice contamination was removed by sublimation. The specimen was then recooled to below −120°C before being returned to the specimen stage of the Gatan Alto 2500 cryo-preparation system at −180°C, where it was coated with about 10 nm of 60:40 gold-palladium alloy (Testbourne Ltd., Basingstoke, UK) in an argon gas atmosphere. The coated specimen was returned to the cold stage of the SEM and examined at a temperature of <–160°C, a beam accelerating voltage of 2 kV, and a beam current of 10 μA, at working distances of 12 to 15 mm. Digital images were captured at a resolution of 2,560 by 1,920 pixels by 8 bits using the signal from the lower secondary electron detector and were saved in TIFF format.

### Live-cell imaging.

**Confocal imaging.** Fertilization events were imaged in inverted agar samples on a Leica SP8x confocal microscope equipped with argon and supercontinuum white light laser (WLL) excitation sources. The excitation wavelengths were 488 nm (sGFP; argon laser) and 555 nm (RFP;WLL); the emission was collected on HyD detectors for sGFP (standard, 496 to 550 nm) and tdimerRed (standard, 600 to 721 nm), while a transmitted light photon multiplier tube (PMT) was used for bright-field collection. The acquisition settings were as follows. For Fig. 2, 6 and 7 and Movies S1, S2, S6, and S7, objective, HCX PL APO CS 10×/0.40 dry; 8 stack intervals of 4.3 μm; stacks aquired every 2 mins 27.4 secs. For Fig. 4 and 5 and Movies S3a and b, S4, S5, S8, and S9, objective, HCX PL APO UVIS CS2 63×/1.20 water. For Movie S3a, 31 stack intervals of 0.7 μm; stacks aquired every 1 min. For Movie S3b, 18 stack intervals of 1 μm; stacks aquired every 9.9 secs. For Fig. 5 and Movie S5, 16 stack intervals of 0.7 μm; stacks aquired every 4.5 secs. For Movie S9, 28 stacks intervals of 1 μm; stacks aquired every 5.4 secs. For female nuclear speed measurements, nuclei were segmented into spots with IMARIS software (Oxford Instruments, UK), and the track speeds for each spot were extracted.

Except for Fig. 1 and 4 and Movie S4, all the pictures and movies were generated with IMARIS as Z-projections with maximum intensity. Fig. 2, 4, 5, and 6 were done using the function “montage” of FIJI software (https://imagej.net/Fiji) (55). For Fig. 4 and Movie S4, wide-field imaging was performed on a temperature-controlled motorized Nikon Te2000 microscope equipped with a Nikon PlanApo VC water immersion 60×/1.2 NA lens objective. Fluorescence images were captured on a Hamamatsu Orca-ER charge-coupled device (CCD) camera (Hamatsu Photonics UK Ltd., UK) under pE-1 LED illumination (CoolLED, UK) exciting at 470 and 550 nm for sGFP and tdimerRed, respectively, using a Semrock GFP/DsRes-2x-A-NTE filter cube (Chroma) and MetaMorph software (Molecular Devices). Four-dimensional (4D) time-lapse stacks were acquired with 3 slices at a stack interval of 1 μm with a frame rate of 29.6 sec^−1^

### Statistical analysis.

A two-way analysis of variance (ANOVA) with fixed effects for target site (hyphae versus trichogyne), time of observation (before versus after nuclear male entry), and their interaction was carried out on all available data. Multiple observations from identical loci were pooled using the arithmetic mean, and data were log-transformed to mitigate the right tailed distribution and to stabilize the variance. *Post hoc* tests were corrected for multiple comparison using the Holm stepwise procedure to ensure an adequate family-wise error rate. A type I error rate of 0.05 was considered for all statistical analyses.
